# Identification of Gene Signature-Related Oxidative Stress for Predicting Prognosis of Colorectal Cancer

**DOI:** 10.1155/2023/5385742

**Published:** 2023-02-07

**Authors:** Xiaolong Wang, Liang Chen, Hongtao Cao, Jianpeng Huang

**Affiliations:** ^1^Department of Gastrointestinal Surgery, Affiliated Huadu Hospital, Southern Medical University (People's Hospital of Huadu District), Guangzhou 510800, China; ^2^Department of Gastrointestinal Surgery, Shenzhen Third People's Hospital, Shenzhen 518100, China

## Abstract

**Background:**

Colorectal cancer (CRC) is the third most common cancer. Nearly a decade of studies had shown that cancer regimens tailored to molecular and pathological features lead to improved overall survival. Oxidative stress (OS) refers to a state in which oxidation and antioxidant effects are unbalanced in the body. However, the molecular mechanism of OS-related CRC remains unclear.

**Methods:**

Univariate Cox regression analysis gained OS signature genes related to CRC prognosis, and then, different CRC molecular subtypes were obtained by consensus clustering analysis. Differential expression analysis and least absolute shrinkage and selection operator (LASSO) algorithm were used to obtain prognostic-related signature genes. Significantly, risk score was calculated by RiskScore = *Σβi* × Exp*i*. Moreover, the Kaplan-Meier survival analysis, immune cell infiltration, and sensitivity to treatment regimens were performed to assess the model's validity and adaptability. Finally, RiskScore incorporated clinicopathological features to further improve prognostic models and survival prediction.

**Results:**

63 OS-related prognostic genes were obtained, and four distinct molecular subtypes of CRC were identified based on the expression characteristics. 230 differentially expressed genes (DEGs) between different molecular subtypes were compressed by LASSO algorithm, and finally, 6 OS-related genes were obtained. The Kaplan-Meier survival analysis indicated that the high RiskScore groups had poorer prognosis and the RiskScore model showed better predictive performance in all three other independent datasets. Moreover, immunotherapy/chemosensitivity analysis found that the low-risk group was more sensitive to different treatment options and could achieve better treatment outcomes.

**Conclusion:**

Oxidative stress-related RiskScore model built in this work has good predictive performance for CRC.

## 1. Introduction

Colorectal cancer (CRC), characterized by blood in the stool, weight loss, and tiredness, is a cancer with the highest incidence [1]. Although the implementation of CRC screening such as fecal occult blood testing and endoscopy has brought promising prospects for CRC cure and greatly improved overall survival, the CRC still maintains a higher death rate [2–4]. Of new CRC diagnoses, 20% of patients have metastatic disease at presentation and the incidence of CRC accounts for 65% of the total incidence [5]. Lots of CRC cases pose a growing challenge to global public health; hence, raising awareness of CRC is significant for promoting better lifestyle quality.

Oxidative stress (OS), specifically the imbalance of oxidative and antioxidative effects in the body, has been documented to be associated with neurodegenerative diseases, cardiovascular disease, diabetes, and many other conditions [6]. OS results in inflammatory infiltration of neutrophils and is considered an important factor in aging and disease. Oxidative stress can be assessed directly by measuring reactive oxygen species (ROS) or indirectly by the associated damage to lipids, proteins, and nucleic acids caused by overproduced ROS [7]. Cancer cells exhibit abnormal redox homeostasis, but although ROS are tumor-promoting, high ROS levels are cytotoxic [8]. In CRC, ROS can mediate genetic changes [9]. Studies have shown that OS was a risk factor for CRC and that malondialdehyde and 4-hydroxy-2-nonenal levels increased significantly in CRC with its stage [10]. Although a recent study showed that OS was closely associated with CRC, the OS-related CRC prognostic key genes were not carefully explored in that work [11].

Tumor immunotherapy is based on the theory that cancer growth is monitored by the immune system [12]. The main goal of immunotherapy is to restore the immune function of antitumor cells and thus improve the ability of the immune system to recognize and eliminate malignant cells [13]. In immune therapy, there are a variety of different categories of drug therapy, which consists of cytokines, immune regulatory monoclonal antibody (immunomodulatorymonoclonal antibodies (mAb)), monoclonal antibody targeting tumor and cancer vaccines, soluble tumor viruses, and adoptive cell therapy. Checkpoint inhibitors are immunomodulators that have revolutionized cancer treatment strategies and have become the standard of care for a variety of tumors [13, 14]. Effective immunotherapy mainly depends on the immune status of tumor microenvironment (TME), and if the tumor contains high density of tumor infiltrating lymphocytes (TILs), it may be more effective for immunotherapy [15, 16].

Here, in this work, based on OS-related genes, we identified stable molecular subtypes by consensus clustering and further explored the characteristics of different subtypes in detail. Finally, we identified OS-related signature genes through differential expression analysis and least absolute shrinkage and selection operator (LASSO) regression analysis. Moreover, we constructed a RiskScore model and a clinical prognosis model, which can assist colorectal cancer patients and be used for personalized treatment of colorectal cancer patients.

## 2. Materials and Methods

### 2.1. Data Collection

We used TCGA GDC API to download gene expression profiling data for TCGA-COAD and TCGA-READ. TCGA-COAD obtained 386 samples, and TCGA-READ obtained 146 samples. Further, the expression data of GSE39582 and GSE87211 data were obtained from the Gene Expression Omnibus (GEO) database, and 562 and 197 samples were finally obtained [17, 18]. In this study, we used GSE39582 as the training set and TCGA-COAD, TCGA-READ, and GSE87211 datasets as independent validation sets.

### 2.2. Data Preprocessing

The gene expression data (fragments per kilobase of transcript per million fragments mapped (FPKM)) of TCGA was preprocessed with rigorous screening steps. Samples without clinical follow-up information, survival time, and survival status were removed. Then, Ensembl was converted to gene symbol. Finally, the expression with multiple gene symbols takes the mean value.

For the GEO dataset, we downloaded the annotation information of the corresponding chip platform, mapped probes to genes according to the annotation information, and removed probes that matched one probe to multiple genes. When multiple probes matched a gene, the median was taken as the gene expression value.

### 2.3. Acquisition of Oxidative Stress-Related Gene

The 436 oxidative stress-related genes in this study were download from the “GOBP_RESPONSE_TO_OXIDATIVE_STRESS” pathway in the MSigDB database [19].

### 2.4. Molecular Subtypes of Oxidative Stress-Related Genes

Different molecular subtypes within a sample can be identified and differentiated by a consensus clustering algorithm [20]. Molecular subtypes of the samples were obtained using the expression data of genes related to cellular senescence. We utilized the *pam* algorithm and *canberra* as the metric distance and performed 500 bootstraps, each bootstrap process including 90% of the training set patients. The optimal classification was determined by calculating the agreement matrix and the agreement cumulative distribution function to obtain the molecular subtype of the sample. Notably, the number of clusters is set to be 2 to 10.

### 2.5. The Construction of RiskScore Model

First, the differentially expressed genes (DEGs) between different molecular subtypes were identified, and the threshold was set as |log fold change| > 1 and *p* value < 0.05. Further, the number of genes was further reduced by the method of LASSO regression analysis, and the signature genes associated with OS phenotypes were obtained. Finally, we calculated the risk score for each patient using the following formula:

(1) $$\begin{array}{c} \text{RiskScore}=\sum \beta i\times \text{Exp}i, \end{array}$$

where *i* refers to the gene expression level of the oxidative stress phenotype prognosis-related gene signature and *β* is the signatue gene. According to whether RiskScore > 0, the patients were divided into different RiskScore groups. The Kaplan-Meier algorithm is used to calculate survival differences between different groups.

### 2.6. Gene Set Enrichment Analysis (GSEA)

In order to gain insight into the signaling pathway mechanism between different molecular subtypes, we used GSEA (R package) for pathway analysis; here, we performed GSEA using all candidate gene sets in the Hallmark database [21, 22]. The ferroptosis pathway is derived from the MSigDB database “WP_FERROPTOSIS”; autophagy pathway was derived from MSigDB database “GOBP_REGULATION_OF_AUTOPHAGY” [19]; inflammatory signature-related gene set was downloaded from Liu et al. [23]; angiogenesis-related gene set was obtained from Masiero et al. [24]. FDR < 0.05 was considered a significant enrichment.

### 2.7. Immune Infiltration Analysis

The CIBERSORT algorithm was chosen to quantify the relative abundance of each cell infiltrate in immune microenvironment [25]. We obtained from Charoentong et al.'s research, a gene set marking each tumor immune microenvironment (TIME) infiltrating immune cell type includes activated CD8+ T cells, activated dendritic cells, macrophages, natural killer T cells, and regulatory T cells [26]. Single-sample gene set enrichment analysis (ssGSEA) was used to represent the relative abundance of each TIME infiltrating cell in each sample [27]. ESTIMATE (R package) was used to calculate immune score, stromal score, tumor purity, and ESTIMATE score for each sample [28].

### 2.8. Prediction of Responsiveness to Immunotherapy

We used the tumor immune dysfunction and exclusion (TIDE) analysis to assess the influence of immune mutation score on the prediction of clinical responsiveness to immune-checkpoint inhibitors. The TIDE algorithm is a computational method for predicting immune-checkpoint blockade responsiveness using gene expression profiling [29].

### 2.9. Statistical Analysis

All statistics were performed using the R software (https://www.R-project.org). The Kaplan-Meier curves were performed to test for significant differences in overall survival. Univariate and multivariate Cox proportional hazards regression analyses were also performed to understand the relationship between RiskScore and overall survival. Receiver operating characteristic (ROC) analysis was used to evaluate the sensitivity and specificity of RiskScores in predicting prognosis, and the area under the ROC curve (AUC) was an indicator of the accuracy of prognosis.

## 3. Results

### 3.1. Molecular Subtypes Based on Oxidative Stress-Related Genes

**Figure 1:**
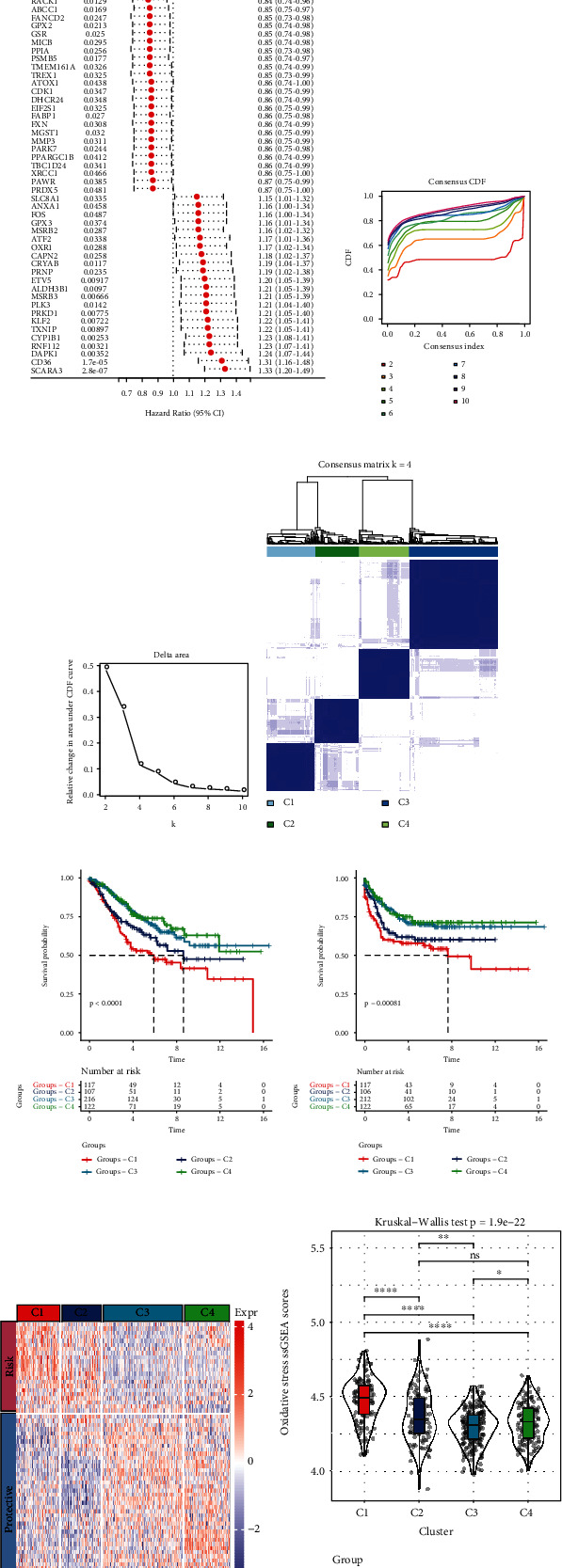
Identification of molecular subtypes based on oxidative stress- (OS-) related genes. (a) Forest plot of prognostic significant OS-related genes. (b) GSE39582 cohort sample cumulative distribution function (CDF) curve. (c) GSE39582 cohort sample CDF delta area curve; delta area curve of consensus clustering, indicating the relative change in area under the cumulative distribution function (CDF) curve for each category number *k* compared with *k*–1. The horizontal axis represents the category number *k*, and the vertical axis represents the relative change in area under CDF curve. (d) The heat map shows the clustering of samples when *k* = 4. (e) The Kaplan-Meier curve shows the overall survival prognosis of the four molecular subtypes. (f) The Kaplan-Meier curve shows the relapse-free survival prognosis of the four molecular subtypes. (g) The heat map showing the expression level of OS-related genes in different molecular subtypes of GSE39582. (h) The violin plot showing differences in “oxidative stress ssGSEA scores” between different molecular subtypes in the GSE39582.

To understand the expression patterns of oxidative stress-related genes, we performed univariate Cox regression analysis using colorectal cancer samples in the GSE39582, and the results showed that 69 oxidative stress genes were associated with colorectal cancer prognosis (*p* value < 0.05) (Figure 1(a)). Patients were classified based on consistent clustering of 69 prognostic oxidative stress gene expression profiles. According to the cumulative distribution function (CDF), the optimal number of clusters is determined. The CDF curve indicated that when *k* = 4, it has a relatively stable clustering result (Figures 1(b) and 1(c)). Finally, 4 molecular subtypes were obtained (Figure 1(d)). The Kaplan-Meier survival analysis, including overall survival and relapse-free survival (RFS), found that C4 has a better prognosis, while the C1 subtype has a worse prognosis (Figures 1(e) and 1(f)). The heat map of the expression trend of oxidative stress genes found that the “risk” gene was highly expressed in the C1 subtype, while the “protective” gene was highly expressed in the C4 subtype (Figure 1(g)). In addition, we also calculated the “oxidative stress ssGSEA scores” of each colorectal cancer patient in the GSE39582 cohort and found that the C1 subtype has a higher score (Figure 1(h)).

### 3.2. Clinical Features among Molecular Subtypes

**Figure 2:**
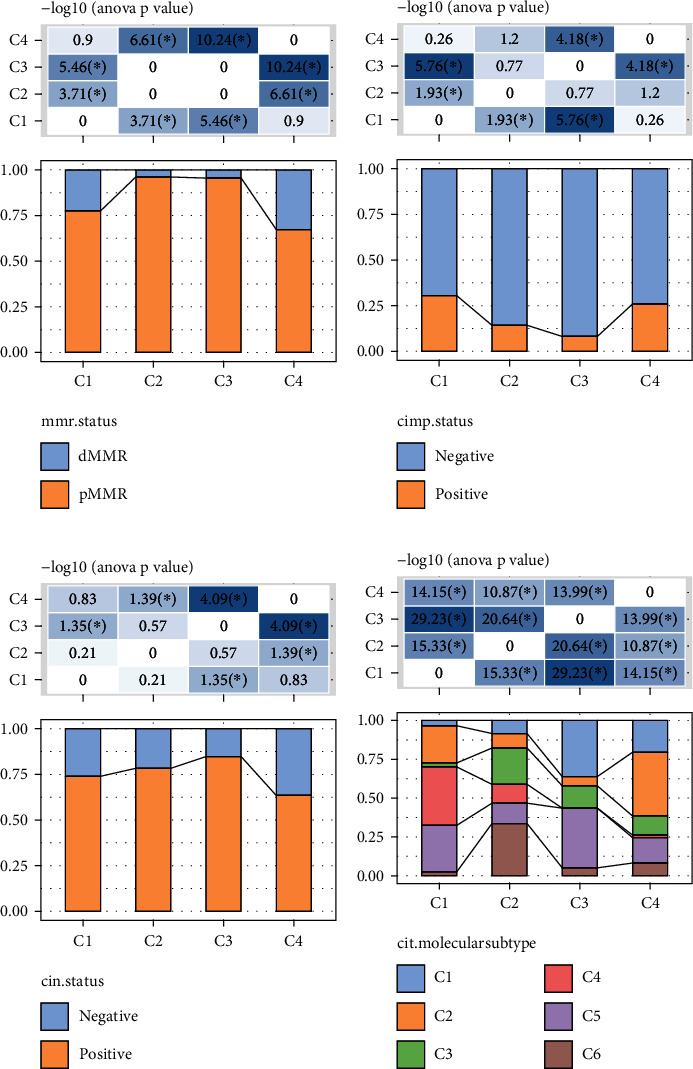
Clinical features among molecular subtypes. (a) Mismatch repair (MMR) status distribution in different clinical features in the GSE39582 cohort. (b) CpG island methylation phenotype (CIMP) status distribution in different clinical features in the GSE39582 cohort. (c) Chromosomal instability (CIN) status distribution in different clinical features in the GSE39582 cohort. (d) Comparative analysis of molecular subtypes in this study and Masiero et al. [24].

Once we divided the molecular subtypes, we attempted to explore differences in clinical characteristics between the different subtypes. A comparative analysis of mismatch repair (MMR) status in different subtypes found that most colorectal cancer patients were pMMR, in which there were significant differences in MMR status between C1 and C2 and between C1 and C3, and the C2 and C3 subtypes were significantly enriched pMMR (Figure 2(a)). In terms of CpG island methylation phenotype (CIMP), there were significant differences in CIMP status between C1 and C2, C1 and C3, and C3 and C4, with C1 and C4 subtypes significantly enriched for positive CIMP status (Figure 2(b)). Chromosomal instability (CIN) analysis suggested that C1, C2, and C3 were significantly with enrichment of CIN positive (Figure 2(c)). We also compared the differences between the molecular subtypes defined in the previous study and our 3 molecular subtypes in this study and found that the C1 subtypes in this study mostly belongs to “C2” “C4” and “C5” subtypes in previous study. The proportion of the “C6” subtype in the C2 subtype in the study was significantly higher than that of other molecular subtypes we defined (Figure 2(d)). Moreover, the results showed that the C1 subtype had a higher clinical grade, but age and sex did not differ significantly among the different subtypes (Figure S1).

### 3.3. Immune Signatures between Molecular Subtypes

**Figure 3:**
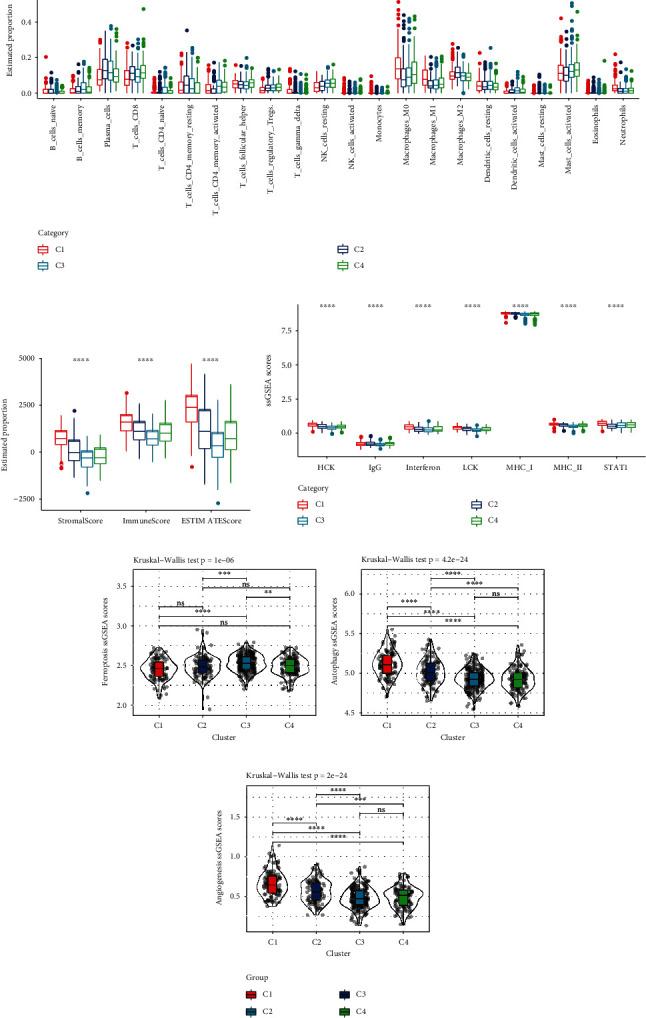
Immune features among molecular subtypes. (a) Differences in 22 immune cell scores between different molecular subtypes in GSE39582 cohort. (b) Differences in immune infiltration among different molecular subtypes in GSE39582 cohort. (c) Inflammation-related gene cluster score differences between different molecular subtypes in GSE39582 cohort. (d–f) Differences in ferroptosis scores, autophagy scores, and angiogenesis scores between different molecular subtypes, respectively.

To further elucidate differences in the immune microenvironment of patients between different molecular subtypes, the extent of immune cell infiltration in patients in the GSE39582 cohort was assessed. Except for activated natural killer (NK) cells, monocytes, and resting dendritic cells, the infiltration degree of other immune cells was different (Figure 3(a)). ESTIMATE analysis showed that the C1 subtype had a significantly higher immune score than other molecular subtypes (Figure 3(b)). Inflammatory activity gene enrichment scores of different molecular subtypes found that 7 different metagenes were significantly different in all four molecular subtypes, and overall C1 and C2 subtypes had higher inflammatory activities (Figure 3(c)). In addition, the ferroptosis score, autophagy score, and angiogenesis score of different molecular subtypes found that the C1 subtype was significantly different from other subtypes (Figures 3(d)–3(f)).

### 3.4. Transcriptional Differences and Identification of Key Genes between Molecular Subtypes

In order to look at the transcription between different molecular subtypes, we identified DEGs between C1 vs. other, C2 vs. other, C3 vs. other, and C4 vs. other subtypes. Since the C1 subtype has a poor prognosis, we explored the C1 subtype in detail. The volcano plot shows 199 DEGs of C1 vs. other, of which 163 were upregulated and 36 were downregulated (Figure S2A). The heat map showed that these upregulated genes were more highly expressed in the C1 subtype (Figure S2B). Functional enrichment analysis showed that DEG was associated with some immune-related pathways such as cytokine-cytokine receptor interaction and cytokine receptor and Toll-like receptor signaling pathway (Figure S2C).

**Figure 4:**
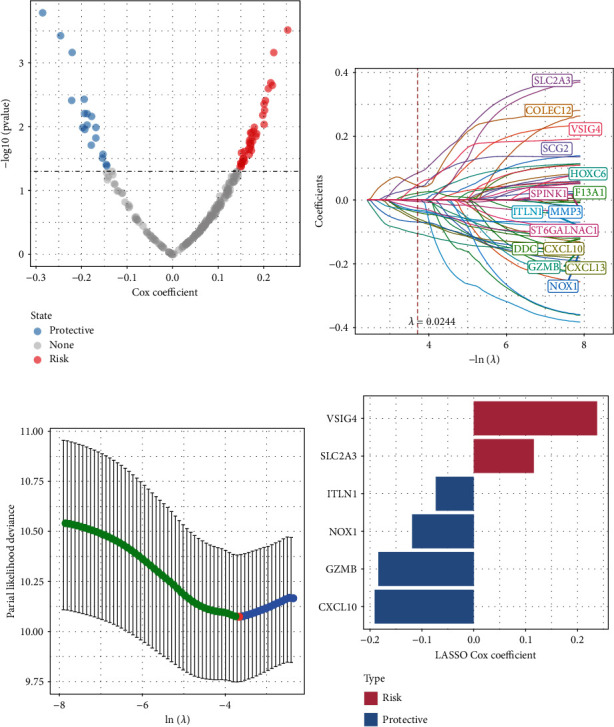
Identification of OS-related prognostic genes. (a) Univariate Cox analysis of DEGs and a total of 53 promising candidates were identified. Red means risk, and blue means protective. (b) The trajectory of each promising candidate genes as a function of lambda. (c) Confidence interval for lambda. (d) Distribution of LASSO coefficients for six genes.

Moreover, univariate Cox regression analysis of DEGs identified a total of 53 genes with a greater impact on prognosis (*p* value < 0.05), including 35 “risk” and 18 “protective” (Figure 4(a)). The results of the LASSO algorithm showed that the model was optimal when lambda = 0.0244 and 15 candidate genes were obtained (Figures 4(b) and 4(c)). We further narrowed down the candidate genes by stepwise multivariate regression analysis and finally obtained 6 oxidative stress-related signature genes (Figure 4(d)). RiskScore = +0.116^∗^SLC2A3 + 0.238^∗^VSIG4 − 0.191^∗^CXCL10 − 0.119^∗^NOX1 − 0.073^∗^ITLN1 − 0.184^∗^GZMB.

### 3.5. Establishment and Validation of RiskScore Model

**Figure 5:**
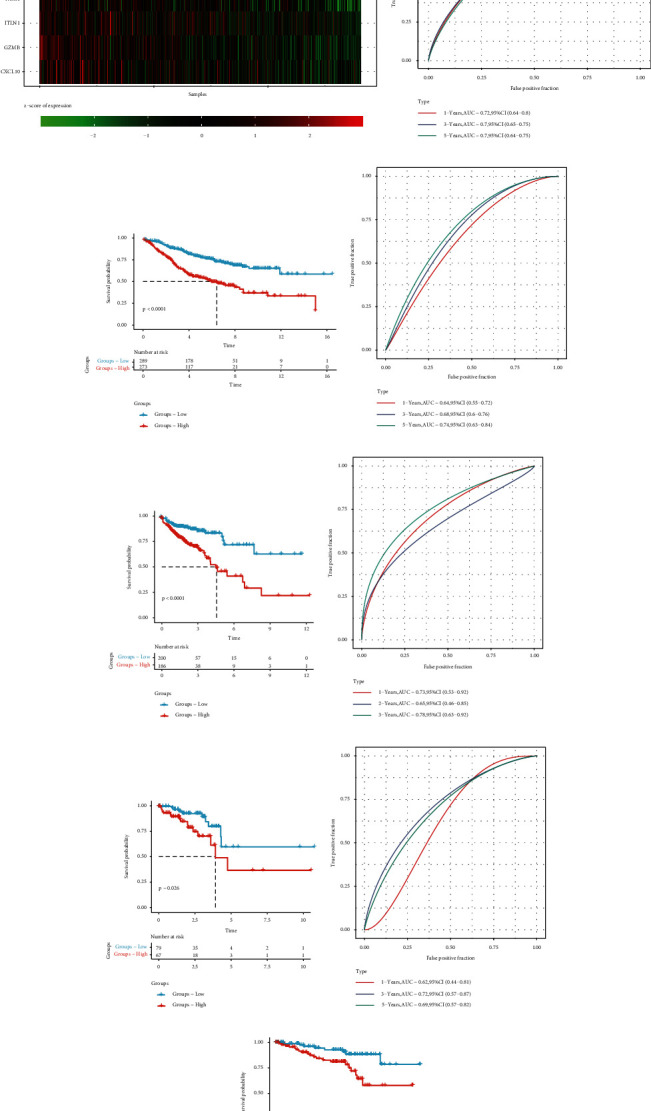
RiskScore model establishment and effectiveness evaluation. (a) The distribution of RiskScore in the GSE39582 cohort: the top panel shows the RiskScore distribution, the middle panel shows the corresponding survival status of each patient, and the bottom panel shows the expression of six OS-related prognostic genes in each patient. (b) ROC curve was used to evaluate the predictive efficacy of the RiskScore model. (c) The Kaplan-Meier survival analysis showing the distribution of survival for different RiskScore groups. (d–i) ROC curve and the Kaplan-Meier survival analysis of different RiskScore group: (d, e) TCGA-COAD cohort; (f, g) TCGA-READ cohort; (h, i) GSE87211 cohort.

First, the risk score in GSE39582 was calculated according to the RiskScore model formula (Figure 5(a)). To assess the predictive efficiency of the prognostic model in 1-, 3-, and 5-year survival, we performed ROC curve analysis. The AUC was 0.72 at 1 year, 0.1 at 2 years, and 0.7 at 3 years (Figure 5(b)). The Kaplan-Meier survival analysis showed that high RiskScore patients had significantly lower OS than low RiskScore patients (*p* value < 0.0001) (Figure 5(c)). To assess the stability of our RiskScore model, we tested it in three independent colorectal cancer cohorts. The AUC was 0.74, 0.78, and 0.69 at 5 years, respectively (Figure 5(d)). Moreover, the Kaplan-Meier survival analysis showed that low RiskScore patients had significantly longer OS, in three different datasets (*p* value < 0.0001, *p* value = 0.026, and *p* value = 0.0077) (Figure 5(e)).

### 3.6. Association of RiskScore with Clinical Features and Molecular Subtypes

To examine the relationship between RiskScore scores and clinical features of colorectal cancer, we analyzed the differences in RiskScore scores between different stage clinical grades in the GSE39582 dataset. The results show that samples with higher clinical stage have higher RiskScore scores (Figure S2A). We also compared the differences in clinicopathological characteristics between the different RiskScore groups in the GSE39582 cohort and found that the high RiskScore group had higher M and T stages (Figure S2B). RiskScore assessments were performed between different clinicopathological groups and found that the low RiskScore group had a favorable prognosis across the different clinical groups (Figure S2C).

### 3.7. Relationship between RiskScore and Immune Cell Infiltration

**Figure 6:**
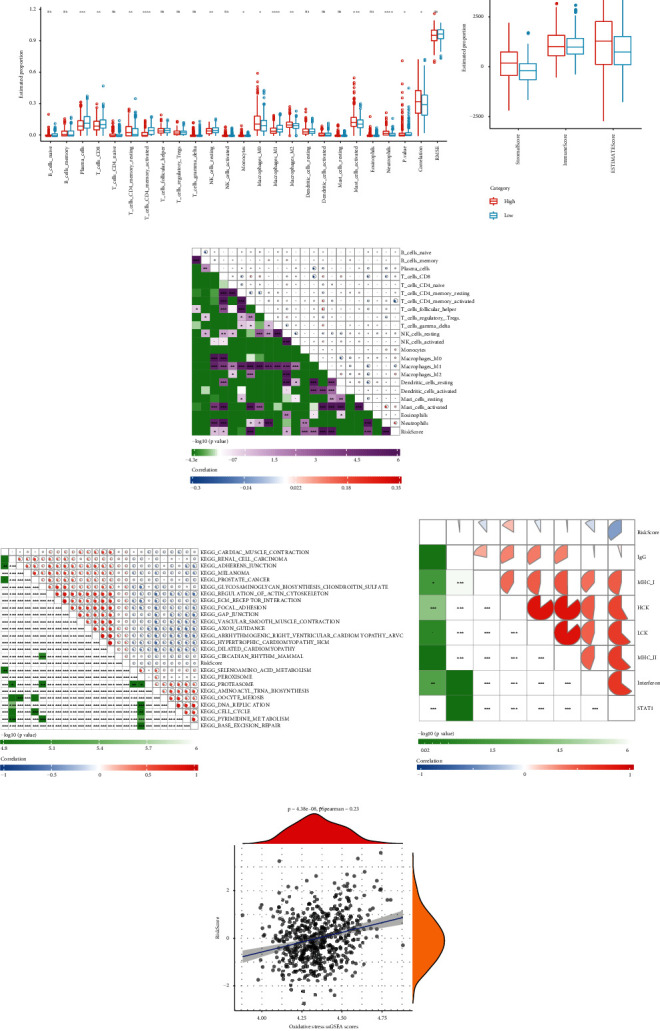
Relationship between RiskScore and immune cell infiltration. (a) Proportion of immune cell components in the GSE39582 cohort. (b) The proportion of immune cell components assessed by ESTIMATE in the GSE39582 cohort. (c) The heat map shows the correlation analysis of 22 immune cells and RiskScore in the GSE39582 cohort. (d) The heat map shows the correlation analysis between signaling pathways and RiskScore in the GSE39582 cohort (*r* > 0.3). (e) The heat map shows the correlation analysis between RiskScore and inflammatory activities in the GSE39582 cohort. (f) The scatter plot shows the correlation between RiskScore and “oxidative stress ssGSEA scores” in the GSE39582 cohort.

To clarify the differences in the immune microenvironment of patients in the RiskScore groups, we evaluated the infiltration degree of 22 immune cells in different RiskScore groups and found that 13 types of immune cells were significantly different in different RiskScore groups (Figure 6(a)). The results of ESTIMATE suggested that stromal score, immune score, and ESTIMATE score were lower in the low RiskScore groups (Figure 6(b)). The correlation analysis between RiskScore and immune cell types indicated that RiskScore was positively correlated with neutrophils and negatively correlated with activated CD4+ memory T cells (Figure 6(c)). ssGSEA suggested that RiskScore was positively correlated with KEGG_CELL_CYCLE and negatively correlated with KEGG_FOCAL_ADHENSION (Figure 6(d)). The correlation analysis between RiskScore and inflammatory activity found that RiskScore was significantly negatively correlated with STAT1 but positively correlated with HCK (Figure 6(e)). In addition, we also compared the correlation between RiskScore and “oxidative stress ssGSEA scores” and found a significant positive correlation (Figure 6(f)).

### 3.8. Relationship of RiskScore and Immunotherapy/Chemotherapy

**Figure 7:**
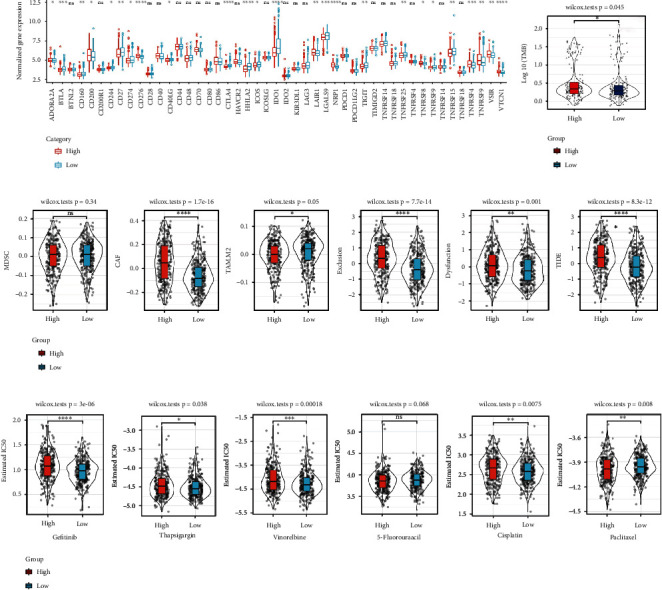
The relationship between immunotherapy/chemotherapy and RiskScore. (a) Box plots showing immune checkpoint expression between different RiskScore groups in the GSE39582 cohort. (b) The difference of tumor mutation burden in the high and low groups. (c) TIDE analysis in the high and low group. (d) The box plots showing IC_50_ for gefitinib, thapsigargin, vinorelbine, 5-fluorouracil, cisplatin, and paclitaxel in GSE39582 cohort.

The results of immune checkpoint expression analysis between RiskScore groups found that the expression of immune checkpoint genes in the low RiskScore group was higher than that in the high RiskScore group. And some immune checkpoint genes are differentially expressed in the RiskScore groups, such as CD276 and IDO1 (Figure 7(a)). The high-risk group had higher tumor mutation burden (Figure 7(b)). The immunotherapy difference analysis found that the TIDE score in the high RiskScore group was higher, suggesting that the high RiskScore group had a higher possibility of immune escape (Figure 7(c)). In addition, the sensitivity analysis of chemotherapy drugs gefitinib, thapsigargin, vinorelbine, 5-fluorouracil, cisplatin, and paclitaxel found that the high RiskScore group was only more sensitive to paclitaxel; however, the low RiskScore group was more sensitive to other chemotherapy drugs (Figure 7(d)).

### 3.9. RiskScore Incorporates Clinicopathological Features to Further Improve Prognostic Models

**Figure 8:**
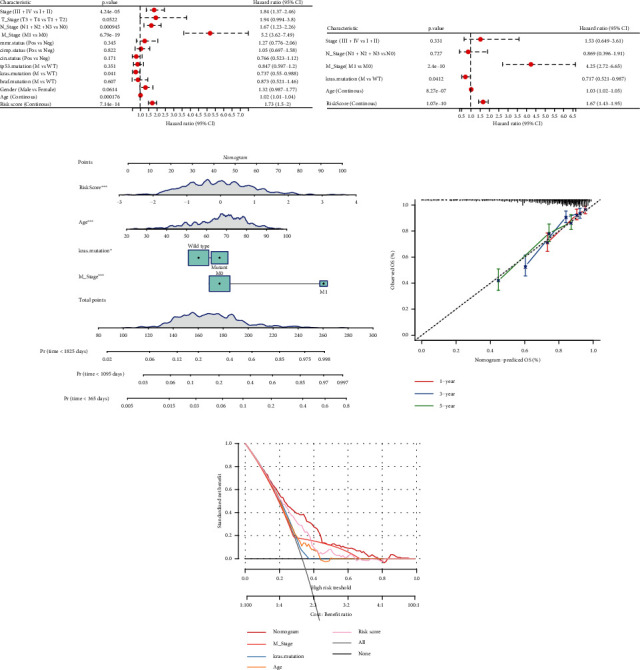
RiskScore incorporates clinicopathological features to further improve prognostic models. (a) Univariate Cox analysis of RiskScore and clinicopathological characteristics. (b) Multivariate Cox analysis of RiskScore and clinicopathological characteristics. (c) Nomogram showing the relationship between RiskScore and clinicopathological characteristics. (d) Calibration curves for 1, 3, and 5 years of nomogram. (e) Decision curve for nomogram.

Univariate and multivariate Cox regression analyses of RiskScore and clinicopathological characteristics showed RiskScore as the most significant prognostic factor (Figures 8(a) and 8(b)). Combining RiskScore and other clinicopathological features to build a nomogram, we found that RiskScore had the greatest impact on survival prediction (Figure 8(c)). Using the calibration curve to evaluate the prediction accuracy of the model, it was found that the predicted calibration curves of the three calibration points in 1, 3, and 5 years were nearly coincident with the standard curve (Figure 8(d)). In addition, we also assessed the reliability of the model using decision curve and found that both nomogram exhibited the strongest survival predictor compared to other clinicopathological features (Figure 8(e)).

## 4. Discussion

Recent developments in the molecular mechanisms of the OS have led to renewed interest in gaining a deeper understanding of CRC [1, 30]. Moreover, interpreting cancer as a genetic disease has gradually been replaced by an imbalance of OS [31]. Studies have shown that genetics, diet, radiation, infection with cytomegalovirus, and genetic polymorphisms of OS-related genes are all related to the development of CRC [32, 33]. Accumulating evidence suggests that OS-related genes play key roles in CRC. Here, based on OS-related genes, through consensus clustering, we identified four stable molecular subtypes with distinct prognostic, pathological, pathway, and immune signatures. Finally, a total of 6 signature genes related to OS phenotypes were screened, and a RiskScore model was constructed, which showed stable prediction performance in independent data sets.

Studies have reported that different molecular subtypes are associated with prognosis in CRC patients [34]. To understand the expression patterns of OS-related genes in CRC patients, first, we obtained 69 OS genes associated with CRC prognosis by univariate Cox regression analysis. Further, we used consensus clustering to classify CRC patients into four distinct molecular subtypes based on OS gene expression patterns. The analysis of clinical characteristics among the four subtypes showed that C4 subtype with better prognosis had higher early clinical characteristics, which also verified the rationality of the four subtypes. A previous study showed that CRCs are classified into six distinct molecular subtypes based on overall gene expression, whereas the present study classified them into four distinct molecular subtypes based on OS-related gene expression profiles [17].

Univariate Cox regression analysis, LASSO regression analysis, and stepwise multivariate regression analysis were used to identify 6 OS prognostic signature genes, including VSIG4, SLC2A3, ITLN1, NOX1, GZMB, and CXCL10. Further, a RiskScore model was constructed. Previous studies suggested that these OS signature genes were involved in regulating CRC development. For example, one study indicated that VSIG4 may regulate macrophage polarization in CRC [35]. Moreover, a recent study reported that upregulation of SLC2A3 in CRC tends to generate poorer prognosis [36]. SLC2A3 mRNA levels were significantly higher in CRC tissues than in adjacent nontumor tissues, which was consistent with our findings [37]. Interestingly, numerous reports confirmed that CXCL10 was a tumor marker in CRC. Previous studies reported that CXCL10 promoted the adhesion of metastatic cells to laminin and thus may antagonize the antitumor effects of chemokines on the tumor microenvironment [38]. CXCL10 had also been elucidated as a marker of CRC liver metastases and poor prognosis [39–41].

Tumor tissue is not simply composed of tumor cells; it is composed of various types of cells, including stromal cells, fibroblasts, and immune cells, which constitute the immune microenvironment of the tumor [42]. A large number of studies have reported that the immune microenvironment of tumors was closely related to the development of tumors [43, 44]. The immune infiltration analysis indicated that reduced immune cell infiltration in the tumor microenvironment in the high RiskScore groups may contribute to poorer patient outcomes.

Immunotherapy analysis and chemotherapeutic drug sensitivity analysis in different RiskScore groups also were used to assess the efficiency of RiskScore model [45]. Excitingly, the low RiskScore group was more sensitive both in response to immunotherapy and chemotherapy, which may be one of the reasons for the better prognosis.

Moreover, the predictors of survival in multivariate analysis of CRC patients were mainly related to RiskScore, nomogram, and age. Tumor stage and chemotherapy have been previously shown to be independent prognostic factors after surgery in CRC patients [46, 47].

In these studies, we found that the predictive value of a single indicator is limited, while the predictive value of a combination of indicators is higher. Therefore, RiskScore and nomogram were constructed to predict CRC prognosis. This is a new predictive model and has been proven effective. However, this work also has limitations. This paper only considers the impact of OS-related genes on CRC from the transcriptional level, and some other factors, such as environment, diet, genetics, and gene modification, are not considered.

## 5. Conclusion

We classified CRC into four distinct molecular subtypes based on OS characteristics. The RiskScore model established in this study is based on OS-related signature genes. A high RiskScore was found to be a strong indicator of poor prognosis. The model shows good performance in different datasets.

## Data Availability

The datasets generated and/or analyzed during the current study are available in the GSE39582 repository (https://www.ncbi.nlm.nih.gov/geo/query/acc.cgi?acc= GSE39582) and GSE87211 repository (https://www.ncbi.nlm.nih.gov/geo/query/acc.cgi?acc= GSE87211).

## Supplementary Materials

Supplementary 1 Fig S1: relationship of different clinical features to molecular subtypes, including T stage, N stage, M stage, stage, age, gender, TP53 mutation status, KRAS mutation status, and BRAF mutation status.

Supplementary 2 Fig S2: identification of differentially expressed genes (DEGs) between molecular subtypes. (A) Volcano plot showing DEGs of C1 vs. other in the GSE39582 cohort. Red means upregulation DEGs, and blue means downregulation DEGs. (B) Heat map showing the expression levels of DEGs of C1 vs. other in the GSE39582 cohort. (C) The functional enrichment analysis of DEGs, including Gene Ontology (GO) and Kyoto Encyclopedia of Genes and Genomes (KEGG) pathway analyses. The size of the dot represents the number of genes, and the color represents the FDR. FDR < 0.05 was considered significant.

Supplementary 3 Fig S3: the efficiency of RiskScore model in clinical features and molecular subtypes. (A) RiskScore distribution in different clinical features in the GSE39582 cohort. (B) Clinicopathological characteristics between RiskScore groups in the GSE39582 cohort. (C) The Kaplan-Meier survival analysis between high and low RiskScore groups in different clinicopathological groups in the GSE39582 cohort.
