# Supplementary material for: Identification of Gene Signature-Related Oxidative Stress for Predicting Prognosis of Colorectal Cancer
Source: Oxid Med Cell Longev. 2023 Feb 7;2023:5385742. doi: 10.1155/2023/5385742 (PMC9936508; doi:10.1155/2023/5385742)
Supplement: Supplementary 1 — Fig S1: relationship of different clinical features to molecular subtypes, including T stage, N stage, M stage, stage, age, gender, TP53 mutation status, KRAS mutation status, and BRAF mutation status. [file 5385742.f1.pdf]

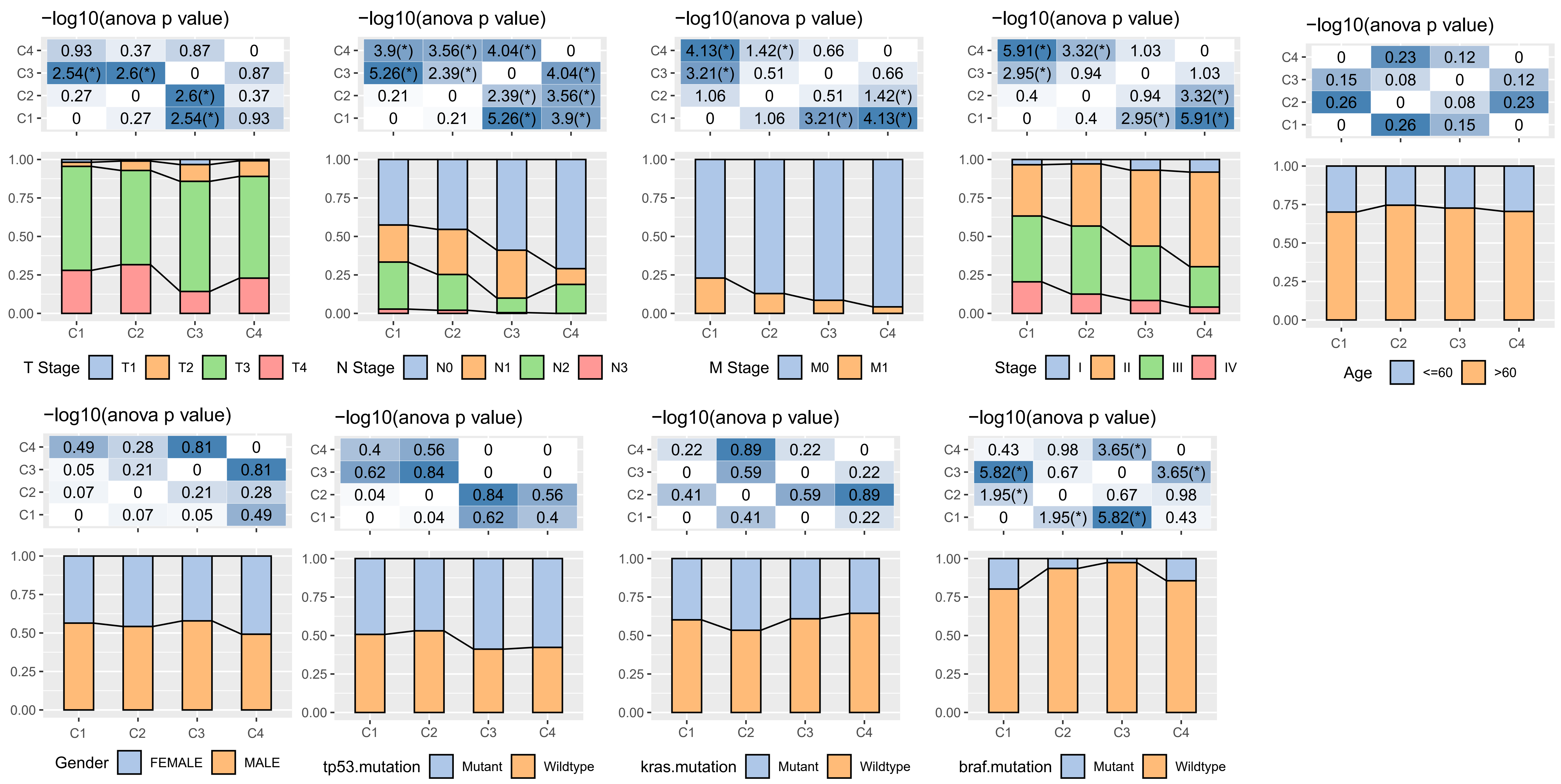

−log10(anova p value)
C4 0.93 0.37 0.87 0
C3 2.54(*) 2.6(*) 0 0.87
C2 0.27 0 2.6(*) 0.37
C1 0 0.27 2.54(*) 0.93
T Stage T1 T2 T3 T4
−log10(anova p value)
C4 3.9(*) 3.56(*) 4.04(*) 0
C3 5.26(*) 2.39(*) 0 4.04(*)
C2 0.21 0 2.39(*) 3.56(*)
C1 0 0.21 5.26(*) 3.9(*)
N Stage N0 N1 N2 N3
−log10(anova p value)
C4 4.13(*) 1.42(*) 0.66 0
C3 3.21(*) 0.51 0 0.66
C2 1.06 0 0.51 1.42(*)
C1 0 1.06 3.21(*) 4.13(*)
M Stage M0 M1
−log10(anova p value)
C4 5.91(*) 3.32(*) 1.03 0
C3 2.95(*) 0.94 0 1.03
C2 0.4 0 0.94 3.32(*)
C1 0 0.4 2.95(*) 5.91(*)
Stage I II III IV
−log10(anova p value)
C4 0 0.23 0.12 0
C3 0.15 0.08 0 0.12
C2 0.26 0 0.08 0.23
C1 0 0.26 0.15 0
Age <=60 >60
−log10(anova p value)
C4 0.49 0.28 0.81 0
C3 0.05 0.21 0 0.81
C2 0.07 0 0.21 0.28
C1 0 0.07 0.05 0.49
Gender FEMALE MALE
−log10(anova p value)
C4 0.4 0.56 0 0
C3 0.62 0.84 0 0
C2 0.04 0 0.84 0.56
C1 0 0.04 0.62 0.4
tp53.mutation Mutant Wildtype
−log10(anova p value)
C4 0.22 0.89 0.22 0
C3 0 0.59 0 0.22
C2 0.41 0 0.59 0.89
C1 0 0.41 0 0.22
kras.mutation Mutant Wildtype
−log10(anova p value)
C4 0.43 0.98 3.65(*) 0
C3 5.82(*) 0.67 0 3.65(*)
C2 1.95(*) 0 0.67 0.98
C1 0 1.95(*) 5.82(*) 0.43
braf.mutation Mutant Wildtype
0.00 0.25 0.50 0.75 1.00
C1 C2 C3 C4
