# Supplementary figures and images for: Identification of Gene Signature-Related Oxidative Stress for Predicting Prognosis of Colorectal Cancer
Source: Oxid Med Cell Longev. 2023 Feb 7;2023:5385742. doi: 10.1155/2023/5385742 (PMC9936508; doi:10.1155/2023/5385742)

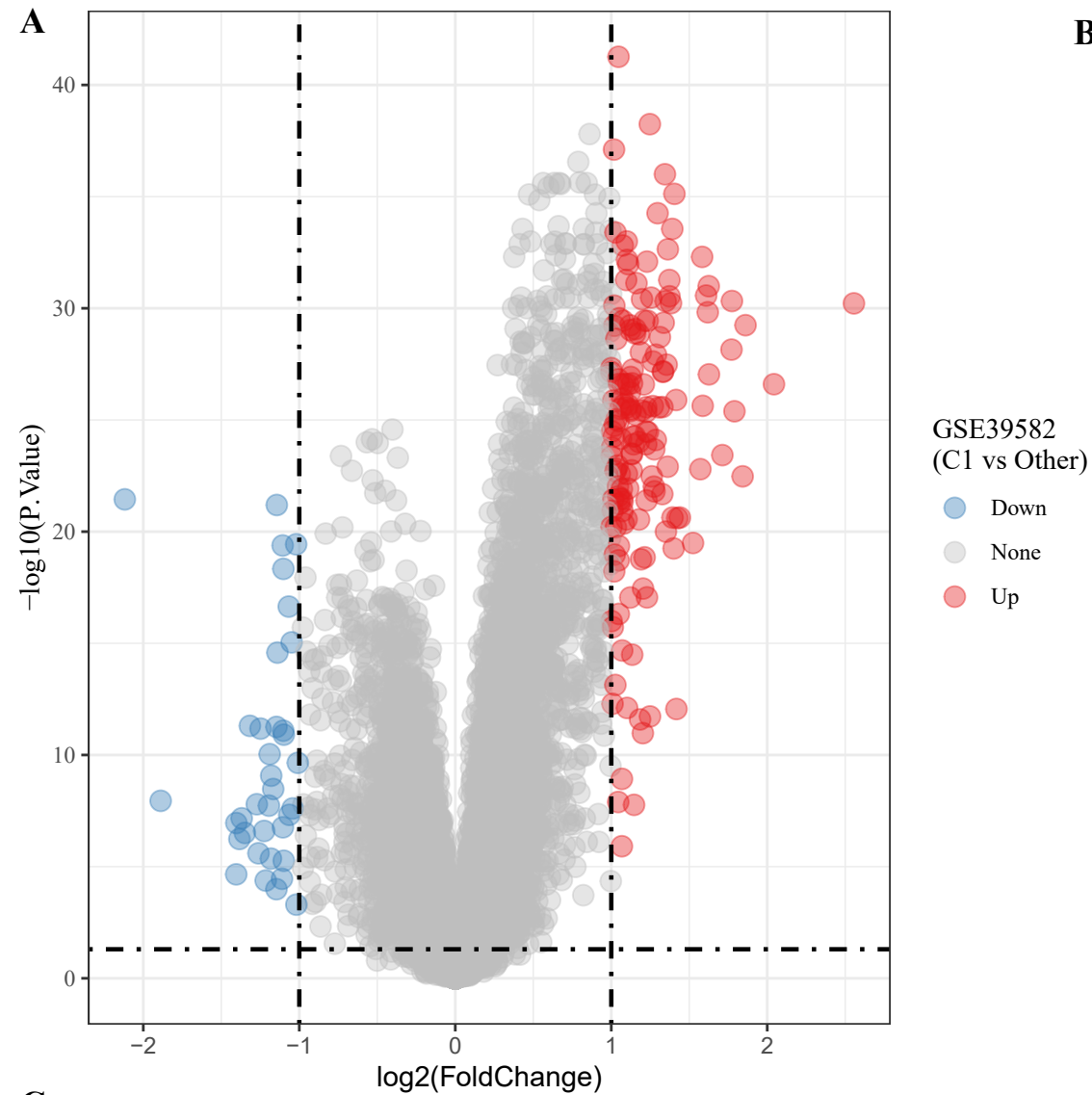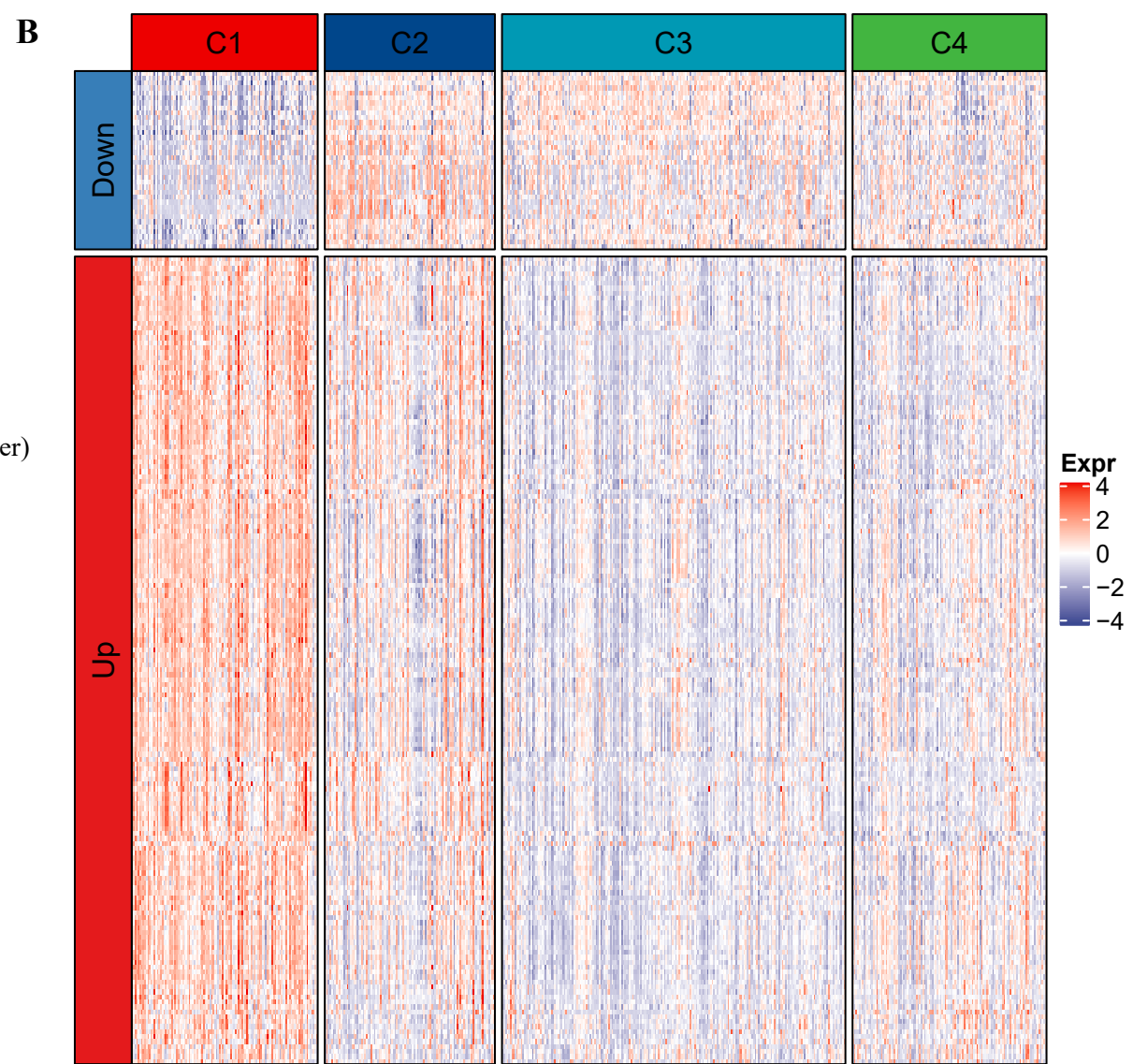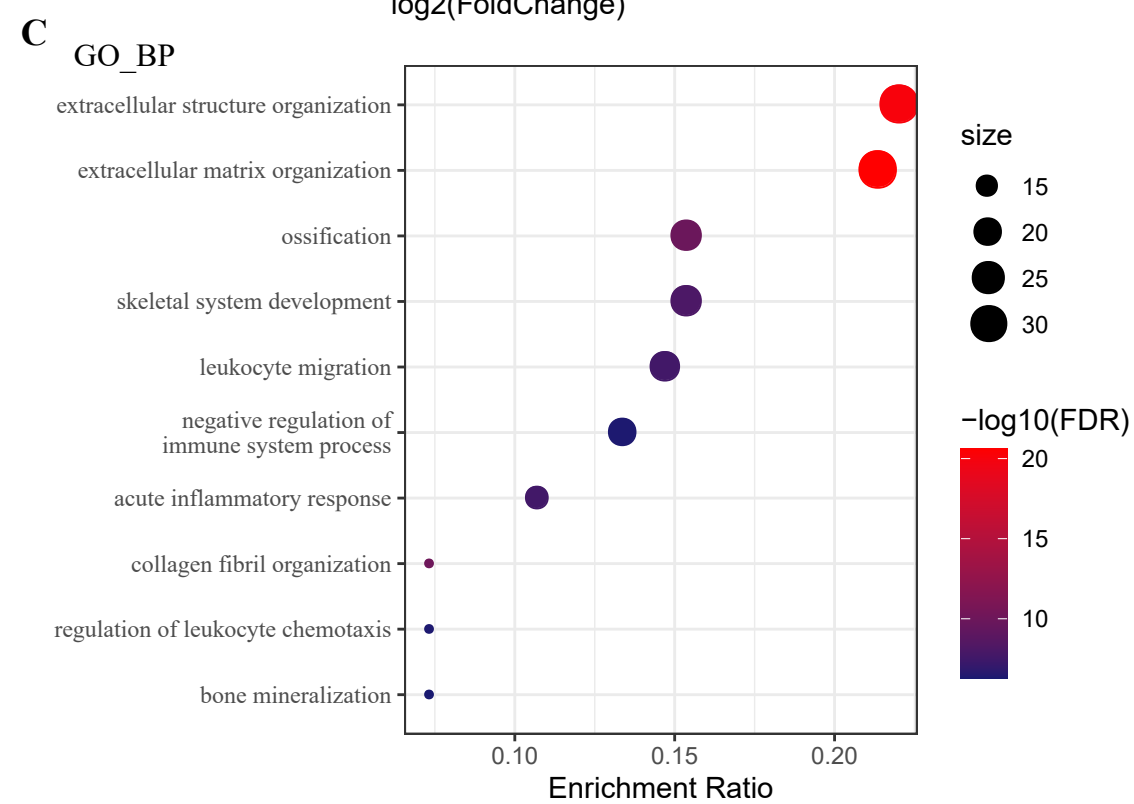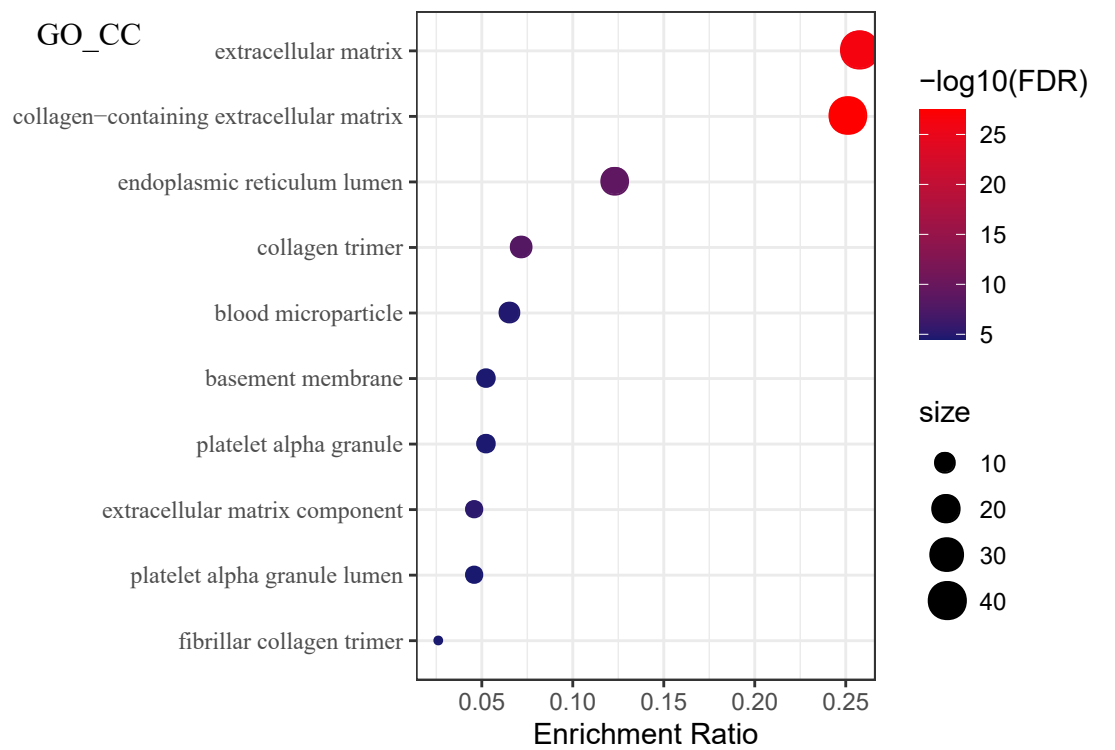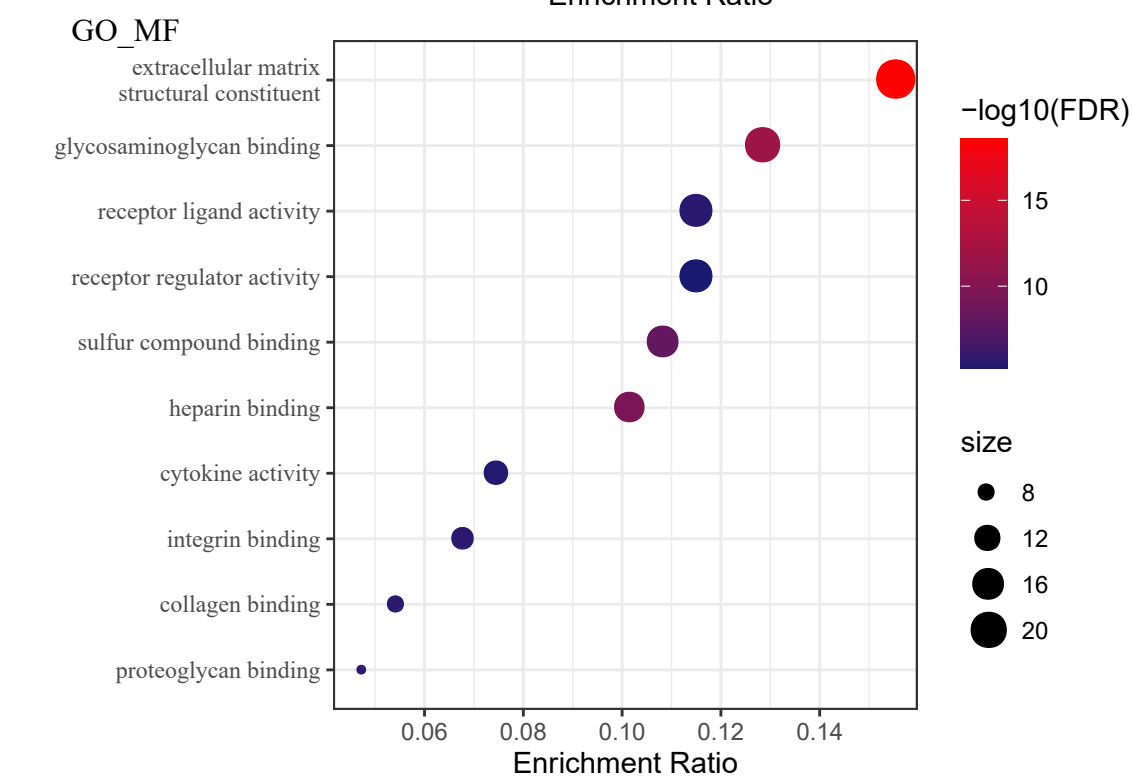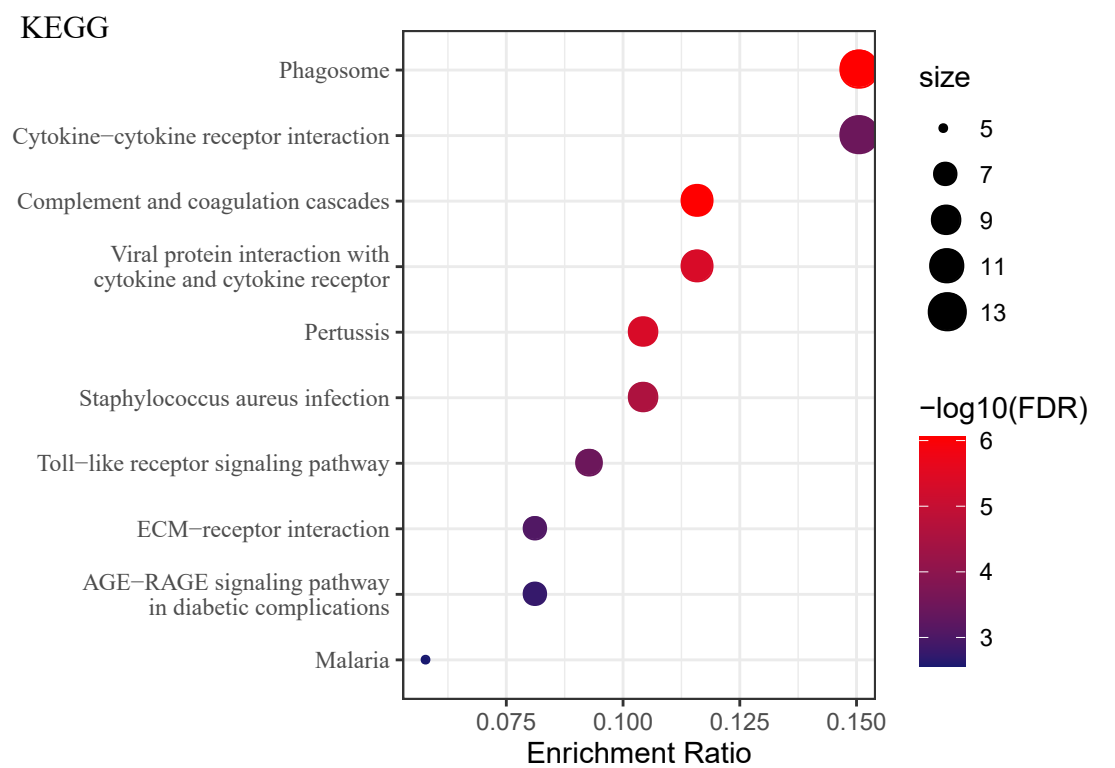

Supplement: Supplementary 2 — Fig S2: identification of differentially expressed genes (DEGs) between molecular subtypes. (A) Volcano plot showing DEGs of C1 vs. other in the GSE39582 cohort. Red means upregulation DEGs, and blue means downregulation DEGs. (B) Heat map showing the expression levels of DEGs of C1 vs. other in the GSE39582 cohort. (C) The functional enrichment analysis of DEGs, including Gene Ontology (GO) and Kyoto Encyclopedia of Genes and Genomes (KEGG) pathway analyses. The size of the dot represents the number of genes, and the color represents the FDR. FDR < 0.05 was considered significant. [file 5385742.f2.pdf]
