# Supplementary material for: Identification of Gene Signature-Related Oxidative Stress for Predicting Prognosis of Colorectal Cancer
Source: Oxid Med Cell Longev. 2023 Feb 7;2023:5385742. doi: 10.1155/2023/5385742 (PMC9936508; doi:10.1155/2023/5385742)
Supplement: Supplementary 3 — Fig S3: the efficiency of RiskScore model in clinical features and molecular subtypes. (A) RiskScore distribution in different clinical features in the GSE39582 cohort. (B) Clinicopathological characteristics between RiskScore groups in the GSE39582 cohort. (C) The Kaplan-Meier survival analysis between high and low RiskScore groups in different clinicopathological groups in the GSE39582 cohort. [file 5385742.f3.pdf]

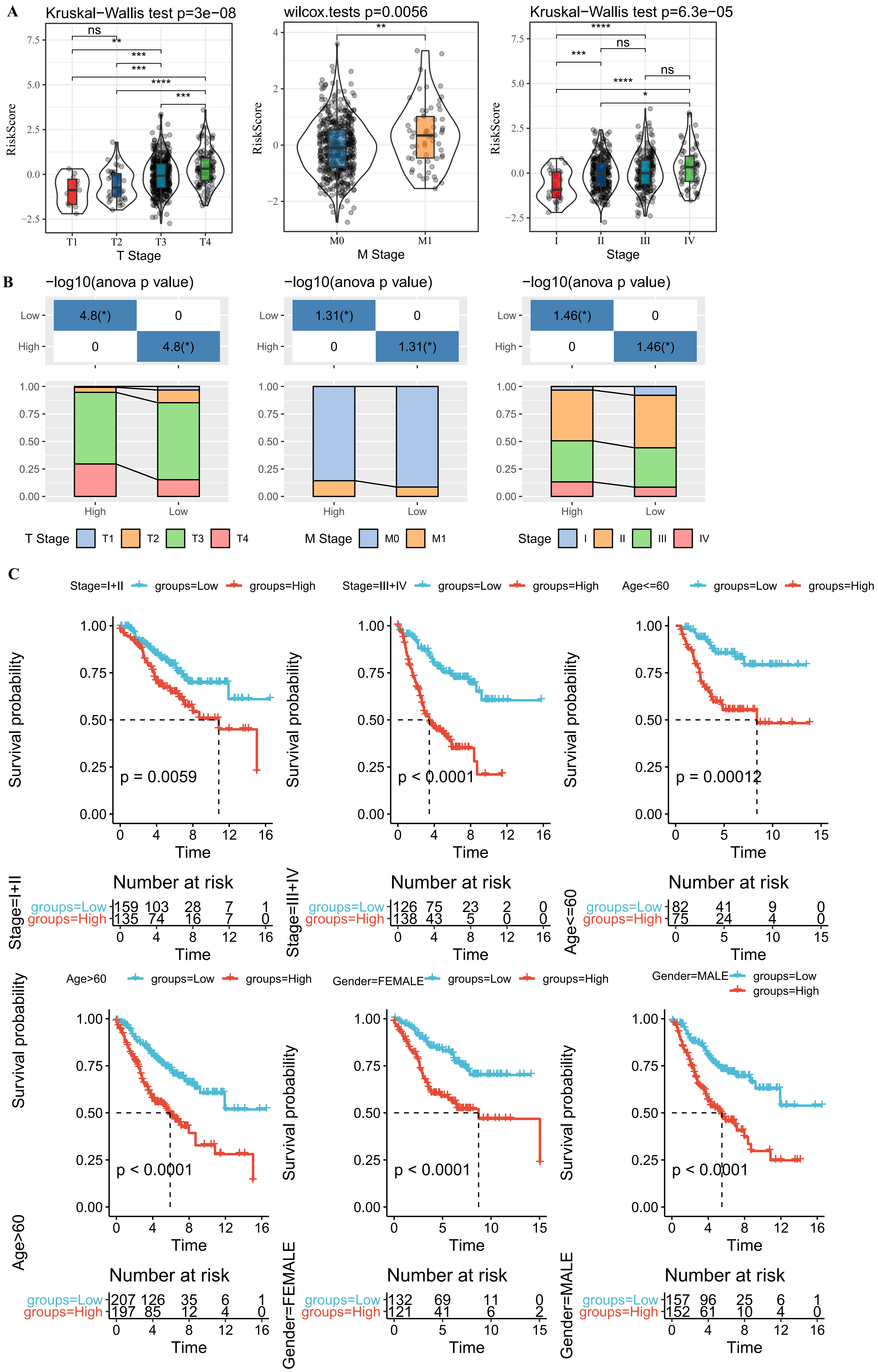
A
Kruskal−Wallis test p=3e−08
RiskScore
T Stage
T1
T2
T3
T4
wilcox.tests p=0.0056
M Stage
M0
M1
Kruskal−Wallis test p=6.3e−05
Stage
I
II
III
IV
B
−log10(anova p value)
Low
High
4.8(*)
1.31(*)
1.46(*)
T Stage T1 T2 T3 T4
M Stage M0 M1
Stage I II III IV
C
Stage=I+II groups=Low groups=High
Survival probability
p = 0.0059
Time
Number at risk
groups=Low 159 103 28 7 1
groups=High 135 74 16 7 0
Stage=III+IV groups=Low groups=High
p < 0.0001
groups=Low 126 75 23 2 0
groups=High 138 43 5 0 0
Age<=60 groups=Low groups=High
p = 0.00012
groups=Low 82 41 9 0
groups=High 75 24 4 0
Age>60 groups=Low groups=High
p < 0.0001
groups=Low 207 126 35 6 1
groups=High 197 85 12 4 0
Gender=FEMALE groups=Low groups=High
p < 0.0001
groups=Low 132 69 11 0
groups=High 121 41 6 2
Gender=MALE groups=Low groups=High
p < 0.0001
groups=Low 157 96 25 6 1
groups=High 152 61 10 4 0
